# Dietary patterns and their associations with gestational weight gain in the United Arab Emirates: results from the MISC cohort

**DOI:** 10.1186/s12937-020-00553-9

**Published:** 2020-04-21

**Authors:** Leila Itani, Hadia Radwan, Mona Hashim, Hayder Hasan, Reyad Shaker Obaid, Hessa Al Ghazal, Marwa Al Hilali, Rana Rayess, Hamid Jan Jan Mohamed, Rena Hamadeh, Hiba Al Rifai, Farah Naja

**Affiliations:** 1 Department of Nutrition and Dietetics, Faculty of Health Sciences, Beirut Arab University, Beirut, Lebanon; 2 Department of Clinical Nutrition and Dietetics, College of Health Sciences, Research Institute of Medical and Health Sciences(RIMHS), University of Sharjah, Sharjah, United Arab Emirates; 3 Nutrition and Dietetics Program, Universiti Sains Malaysia, Kelantan, Malaysia; 4 Sharjah Child Friendly Office, Sharjah, United Arab Emirates; 5 Clinical Nutrition Department, Al Qassimi Hospital-Ministry of Health and Prevention, Sharjah, United Arab Emirates; 6 Department of Nutrition and Food Sciences, Faculty of Agriculture and Food Sciences, American University of Beirut, Beirut, Lebanon

**Keywords:** Gestational weight gain, Gestational weight gain rate, Dietary patterns, Nutrient intake, Social determinants

## Abstract

**Background:**

Suboptimum weight gain during pregnancy may carry long term health consequences for the infant or mother. Nutritional imbalances are well recognized as a determinant of gestational weight gain. Few studies examined the effect of dietary patterns on gestational weight gain, especially in countries undergoing nutrition transition, such as the United Arab Emirates.

**Objectives:**

To characterize dietary patterns among pregnant women living in the UAE and examine their associations with gestational weight gain and gestational weight rate.

**Methodology:**

Data were drawn from the Mother-Infant Study Cohort, a two-year prospective cohort study of pregnant women living in the United Arab Emirates, recruited during their third trimester (*n* = 242). Weight gain during pregnancy was calculated using data from medical records. The Institute of Medicine’s recommendations were used to categorize gestational weight gain and gestational weight gain rate into insufficient, adequate, and excessive. During face-to-face interviews, dietary intake was assessed using an 89-item culture-specific semi-quantitative food frequency questionnaire that referred to usual intake during pregnancy. Dietary patterns were derived by principal component analysis. Multiple logistic regression analyses were used to evaluate the associations of derived dietary patterns with gestational weight gain/gestational weight gain rate.

**Results:**

Two dietary patterns were derived, a “Diverse” and a “Western” pattern. The “Diverse” pattern was characterized by higher intake of fruits, vegetables, mixed dishes while the “Western” pattern consisted of sweets and fast food. The “Western” pattern was associated with excessive gestational weight gain (OR:4.04,95% CI:1.07–15.24) and gestational weight gain rate (OR: 4.38, 95% CI:1.28–15.03) while the “Diverse” pattern decreased the risk of inadequate gestational weight gain (OR:0.24, 95% CI:0.06–0.97) and gestational weight gain rate (OR:0.28, 95% CI:0.09–0.90).

**Conclusion:**

The findings of this study showed that adherence to a “Diverse” pattern reduced the risk of insufficient gestational weight gain/gestational weight gain rate, while higher consumption of the “Western” pattern increased the risk of excessive gestational weight gain/gestational weight gain rate. In view of the established consequences of gestational weight gain on the health of the mother and child, there is a critical need for health policies and interventions to promote a healthy lifestyle eating through a life course approach.

## Background

The first one thousand days, including days spent in utero, are considered a critical and sensitive window in the lifespan, during which environmental exposures have a long-lasting impact on the baby’s growth and future health. Emerging evidence suggested that the quality and quantity of the mother’s nutrition during pregnancy is an important modifiable environmental factor that is associated, not only with growth and development of the fetus but also with the early programming of chronic diseases’ development later in life including hypertension, diabetes, cardiovascular disorders, obesity, as well as neuropsychological disorders [1].

Gestational weight gain (GWG) is one of the factors determined by the mother’s nutrition, which is postulated to influence pregnancy outcomes [2]. Evidence has consistently linked inadequate GWG with adverse outcomes for both the baby and the mother. A systematic review and meta-analysis compiled data from one million pregnant women and reported a significant association between excessive GWG and increased risk of delivering a macrosomic and large for gestational age (LGA) babies. On the other hand, insufficient GWG was significantly associated with preterm delivery and small for gestational age (SGA) babies [3]. Such findings are alarming in light of the evidence linking both LGA and SGA with obesity and other chronic diseases later in life [4]. As for the mother, studies have reported associations between excessive GWG and pregnancy hypertensive disorders, diabetes, emergency cesarean delivery, postpartum weight retention, and obesity in women [5–8]. A prospective cohort study reported that excessive GWG was associated with a 47% increased risk of type 2 diabetes compared to weight gain within the recommendations [9]. Another cohort of 12,522 women, found that the risks of gestational hypertensive disorders and preeclampsia were higher among women with excessive GWG compared to women gaining weight within the recommendations [10]. The recent recommendations of GWG have focused on ensuring optimum pregnancy outcomes in terms of mother and infant health [11].

Dietary intake during pregnancy is among the widely investigated environmental exposure, postulated to affect GWG. The latter is reported to be significantly associated with total energy, macronutrients (carbohydrates, fat, and saturated fat), cholesterol, monosaccharides and sucrose, and micronutrients intake, particularly among overweight and obese women [12–14]. Although single-nutrient studies have advanced the understanding of the association between diet and GWG, a more holistic approach to the assessment of diet has recently emerged in nutritional epidemiology, whereby the dietary pattern and not the single-nutrient of the individual is considered as the exposure. This approach overcomes the conceptual and methodological limitations of single-nutrient studies. People eat nutrients and foods in combination, and hence, studying one nutrient/food in isolation is less likely to reveal the true association given the interaction and synergistic effects of these nutrients and foods together [15]. Accordingly, the recent couple of decades have witnessed a plethora of studies examining the association of dietary patterns with health and disease. In the context of GWG, a few studies showed that adherence to a dietary pattern rich in fruits and vegetables during pregnancy was negatively associated with excessive GWG [2, 16, 17]. On the other hand, excessive GWG was found to be more prevalent among women consuming a western type of diet, consisting mostly of meat, fries, salty snack, and dipping sauces [2, 17].

The United Arab Emirates (UAE) is an oil-producing country in the Middle East that has experienced, since the discovery of the oil, a rapid economic growth, increased incomes, globalization of trade and marketing, as well as rapid urbanization. These changes were accompanied by an epidemiological transition, with increasing rates of obesity and other metabolic diseases [18–20]. A recent systematic review assessing the trend in overweight and obesity prevalence has reported a 2–3-fold increase between 1989 and 2017 [21]. Alarmingly, these obesity rates are higher among women (42%) [22]. Such an epidemiological transition has been linked to shifts in dietary intake, whereby western types of diet are increasingly being adopted in the country. Up to date, no studies have examined the association of different dietary patterns on GWG among pregnant women in the UAE. The main objective of this study is to derive and characterize dietary patterns among pregnant women in the UAE and to investigate the associations of these patterns with GWG and gestational weight gain rate (GWGR), using data from the Mother-Infant Study Cohort (MISC). A secondary objective of this study is to examine the correlates of the derived dietary patterns among study participants.

## Methods

Data for this study were drawn from MISC, a two-year prospective cohort study that included 256 pregnant women from the UAE. The study methods and recruitment were described elsewhere [23]. MISC recruited pregnant women in their third trimester, using convenient sampling from prenatal clinics in Dubai, Sharjah, and Ajman in UAE. Participants were interviewed six times (once during pregnancy, at delivery, and 2, 6, 12, and 24 months postpartum) from December 2015 to December 2017. Perinatal information was obtained from hospital records. Over the course of the study, socio-demographic characteristics, lifestyle, dietary intake, anthropometry, infant feeding practices, cognitive development, along with maternal and infant blood profile, and breast milk profile were also obtained.

Written informed consent was obtained from the participants at baseline, and the ethical approvals from the Research and Ethics Committee at the University of Sharjah (REC/14/01/1505), Al Qassimi Clinical Research Centre Ethical Research Committee (REC Reference Number: 21512015 ± 03), Ministry of Health Ethical Research Committee (R02), and Dubai Health Authority (DSREC-0/2016) approved all study protocol. Women were eligible to participate if they met the following criteria: Pregnant Emirati and Arab women in their 3rd trimester (27–42 weeks of gestation), aged 19 to 40 years old; with a singleton pregnancy, free of chronic diseases such as (diabetes, hypertension, kidney disease, and cancer). Women were excluded from participating if they were pregnant with multiple pregnancies or diagnosed as a high-risk pregnancy or had a history of chronic diseases.

For the purpose of this study, data collected during the first visit, as well as information from the medical records were used. The first visit took place in the waiting room of the primary health care centers, where participants were recruited. During this visit, data related to sociodemographic, physical activity, diet, gestational diabetes mellitus (GDM), and anthropometric characteristics were collected. Data was collected using interviewer-administered multi-component questionnaires addressing maternal sociodemographic and lifestyle characteristics such as age (in years), education (intermediate or less, high school/technical diploma and university), employment (employed versus housewife), family monthly income (< 10,000 Arab Emirates Dirham (AED) or > 10,000 AED), nationality (Emirati or Arab), parity (primiparous (one child) or multiparous (more than 1 child), presence of GDM, pre-pregnancy body mass index (BMI) (overweight or normal) and physical activity. The latter was assessed using the Pregnancy Physical Activity Questionnaire (PPAQ) [23], whereby total physical activity was calculated by weighting each type of activity by its energy requirements defined in multiples of the resting metabolic rate for an activity multiplied by the minutes performed (METs-min). Based on METS-min per week, three categories of physical activity were assigned, including low, moderate, and high intensity.

### Maternal dietary intake

Maternal intake was measured using a culture-specific semi-quantitative food frequency questionnaire (FFQ). The FFQ consisted of 89 items and referred to usual dietary intake during pregnancy. For the FFQs, participants were assisted with the referenced portions of the two-dimensional food portion visual (Millenand Morgan, Nutrition Consulting Enterprises, Framingham, Massachusetts, United States). Supplementary visual aids for portion sizes of common items in the traditional Gulf and Middle Eastern cuisine meals [24] were used to help estimate quantities consumed. Daily energy and nutrient intakes were calculated using food composition tables provided by NUTRITIONIST PRO™ diet analysis software (Axxya Systems LLC., USA, version 5.1.0,2014, First Fata Bank, Nutritionist Pro, San Bruno, CA) and the food composition table of Middle Eastern foods for local and traditional dishes [25].

### Gestational diabetes mellitus

Screening for GDM was carried out during 24–28 weeks of gestation using the National Institute for Health and Care Excellence (NICE) Diabetes in Pregnancy criteria [26]. Data were obtained from the clinical record of the participants.

### Maternal pre-pregnancy (BMI)

Maternal pre-pregnancy BMI was calculated using height and pre-pregnancy weight. Mother height was measured during the 1st visit using standard protocol and was measured to the nearest 0.1 cm (cm) using Seca 220 Telescopic Measuring Rod for Column Scales. While the weight before pregnancy was obtained from the medical record. BMI was calculated as weight (in kg) divided by squared height (in meter). Pre-pregnancy BMI was categorized according to the World Health Organization (WHO) classification into 4 categories: underweight (BMI < 18.5 kg/m^2^, normal weight (BMI 18.5–24.9 kg/m^2^), overweight (BMI 25.0–29.9 kg/m^2^) and obese (BMI ≥30.0 kg/m^2^ [25]).

### Assessment of GWG and GWGR

GWG was calculated as the last weight measured before delivery (taken from medical records) minus the pre-pregnancy weight of the mother (also taken from the medical records). GWG was categorized as insufficient, adequate, or excessive weight, taking into account the pre-pregnancy BMI according to the Institute of Medicine’s (IOM) guidelines. These guidelines state that GWG for underweight women ought to be between 12.5 to 18 kg, for women of normal weight between 11.5 to 16 kg, for overweight women between 7 to 11.5 kg, and for obese women between 5 to 9 kg [27] .

GWGR was calculated in function of the maternal pre-pregnancy weight and gestational age at first study visit. As per the IOM criteria [11], GWGR is the rate of weight gain per week of gestation after the 12th week. In this study, total weight gain until the first visit was calculated as the difference between weight at first visit and pregnancy weight. In order to calculate the GWGR in this study, women were assumed to have gained an average of 1 kg during the first trimester. This assumption is based on the IOM weight gain recommendation of 0.5–2 kg over the first trimester [11]. Hence the equation below was used:

1 $$ \frac{GWGR}{week}=\frac{\left({weight}_{v1}- prepregnancy\ weight\right)-1}{calcu\mathrm{l} ated\ gestational\  age-12}, $$

Based on their pre-pregnancy BMI and gestational age at the time of the first visit, the IOM criteria for weight gain per week were used to categorize GWGR. Women were considered to have either insufficient, adequate, or excessive GWGR if their weight gain rate fell below, within or above the recommended weight gain per week, respectively.

### Derivation of dietary patterns

For the purpose of determining the dietary patterns, food items from the FFQ were grouped into 15 food groups based on similarities in ingredients, nutrient profile, and /or culinary usage (see Additional file 1). Food items having a unique composition that differed from other food items, such as eggs, were classified individually. The total daily consumption from each group was calculated as the sum of portion intake of each item in this group. Exploratory Principal Component Factor Analysis (PCFA) with varimax rotation was conducted on the intake of the 15 food groups. The Kaiser–Meyer– Olkin (KMO) test (> 0.6) and the X^2^ Bartlett test of sphericity (*P* < 0.05) were used to examine the sampling adequacy and the strength of correlations between each food group respectively [28]. The number of factors retained was based on the inflection point of the scree plot and the interpretability of factors. Factor loadings indicated the strength and direction of the association between the derived patterns and each food group. A positive loading indicated a direct association between the food group and the pattern, while a negative loading indicated an inverse association. The derived dietary patterns were labeled based on food groups having factor loading greater than 0.4. Each participant received a factor score calculated by a multiple regression approach. These scores indicated the degree to which each subject’s diet corresponds to the identified pattern. Therefore, the higher the factor scores an individual obtained for a certain pattern, the better his/her adherence to this pattern. For each pattern, participants were grouped into quartiles of pattern scores.

### Statistical analyses

Frequencies, means, and standard deviations were used to describe the sociodemographic and anthropometric characteristics of the study participants. Independent sample t-test and chi-square tests were used to compare means and proportions, respectively. Pearson’s correlation coefficient was used to study the association between the derived patterns scores and nutrient intake. Zeigler’s test for dependent samples was used to compare the difference between correlations. Multinomial logistic regression was used to study the association between the derived dietary patterns as independent variables and the weekly rate of GWG as dependent variable. In regression analyses, the adequate GWG and GWGR categories were considered as reference. The models were adjusted for maternal age, pre-pregnancy BMI, parity, presence of GDM, and energy intake. For the multinomial regression only variables with *p*-values < 0.25 in the simple model were included in the final model [29]. The correlates of the dietary patterns were examined using multiple linear regression models where the scores of the patterns were dependent variables, and the various sociodemographic correlates were the independent variables. The Statistical Package for the Social Sciences (SPSS V.25) was used for all computations [30], and a p-value < 0.05 was considered significant.

## Results

During the study period, 256 pregnant women were enrolled. Of those, 245 completed the FFQ. The main reason for not completing the FFQ was time limitation. For three women, weight gain data were missing; hence, they were excluded. Only pregnant women with complete FFQ and with weight gain information were included in this study (*n* = 242). The results of the study showed that 57.4, 18.2, and 24.4% of the MISC participants had excessive, adequate, and insufficient weight gain, respectively. The distribution of the study sample across the categories of GWGR was as follows: 39.3% excessive GWGR, 25.6% adequate GWGR, and 35.1% insufficient GWGR. Table 1 showed the descriptive characteristics of the study sample across the various categories of GWGR. In the study sample, 40.9% of women were Emirati while 59.1% were Arabs. Overall, more than 50% of the participants were older than 30 years of age. Only 12.8% had primary or less education level, with 55.4% having intermediate to high school level. The majority of women were housewives (83.5%). The prevalence of GDM among participants was 19.8%. Almost 60% of women were overweight or obese before pregnancy (BMI ≥ 25 Kg/m^2^). Regarding physical activity, 59.6% of women were in the low-intensity category with only 14.2% belonging to the high-intensity category. The comparison of subjects’ characteristics across categories of GWGR showed that the prevalence of GDM among women with adequate GWGR was significantly lower compared to the rest of the study population (*p* = 0.02). Parity was associated with GWGR where the majority of multiparous had insufficient GWGR (*p* = 0.05).

**Table 1:** Sociodemographic and anthropometric characteristics of the study sample (*n* = 242)

		Gestational weight gain rate (GWGR)			
	Total	Insufficient	Adequate	Excessive	*P*-value^*^
	*n* = 242	*n* = 85	*n* = 62	*n* = 95	
Nationality					0.72
Emiratis	99(40.9)	33(38.8)	28(45.2)	38(40.0)	
Arabs	143(59.1)	52(61.2)	34(54.8)	57(60.0)	
Mother’s age (Years)					
18–24.9	55(22.7)	15(17.6)	12(19.4)	28(29.5)	0.13
25–29.9	61(25.2)	22(25.9)	21(33.9)	18(18.9)	
≥ 30	126(52.1)	48(56.5)	29(46.8)	49(51.6)	
Mother’s education					
Primary or less	31(12.8)	13(15.3)	6(9.7)	12(12.6)	0.73
Intermediate/high school	134(55.4)	43(50.6)	35(56.5)	56(58.9)	
University	77(31.8)	29(34.1)	21(33.9)	27(28.4)	
Mother’s employment					0.79
Housewife/Homemaker	198(83.5)	71(85.5)	51(83.6)	76(81.7)	
Employee	39(16.5)	12(14.5)	10(16.4)	17(18.3)	
Family monthly income					0.23
< 10,000AED	86(47.0)	34(50.7)	17(36.2)	35(50.7)	
> 10,000 AED	97(53.0)	33(49.3)	30(63.8)	34(49.3)	
Parity					
Primiparous	59 (24.4)	15 (17.6)	13 (21.0)	31 (32.6)	0.05
multiparous	183 (75.6)	70 (82.4)	49 (79.0)	64 (67.4)	
Gestational Diabetes Mellitus					0.02
No	194(80.2)	63(74.1)	57(91.9)	74(77.9)	
Yes	48(19.8)	22(25.9)	5(8.1)	21(22.1)	
Pre-pregnancy BMI (Kg/m^2^)					0.15
BMI < 25	97(40.1)	27(31.8)	28(45.2)	42(44.2)	
BMI ≥ 25	145(59.9)	58(68.2)	34(54.8)	53(55.8)	
Physical activity					0.67
Low intensity	130(59.6)	48(59.3)	36(65.5)	46(56.1)	
Moderate intensity	57(26.1)	23(28.4)	13(23.6)	21(25.6)	
High intensity	31(14.2)	10(12.3)	6(10.9)	15(18.3)	

Values in this table represent n (%)

^*^P-values were derived from Chi-square test

**Fig. 1:**
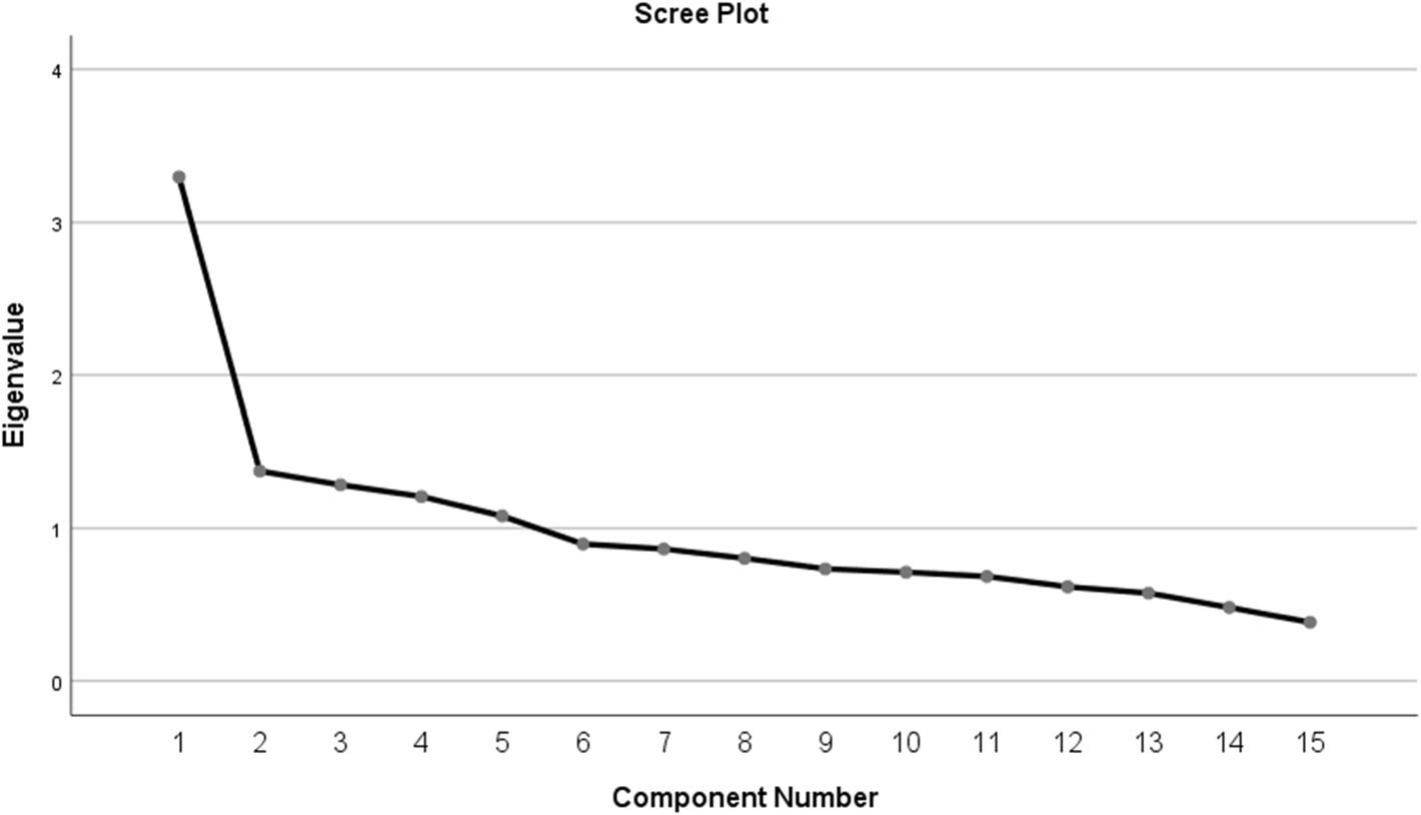
The scree plot showing the inflection point at two factors

PCFA revealed two distinct patterns as indicated by the scree plot (Fig. 1), named as “Diverse” and “Western” dietary patterns, which together explained 31% of the variance in dietary intake in the study population. Table 2 presented the factor loadings of various food groups/items for these patterns. The “Diverse” dietary pattern was characterized by consumption of fruits (0.63), vegetables (0.61), mixed dishes (0.59), meats (0.54), dairy (0.49), grains (0.48), legumes and nuts (0.46), as well as fats and oils (0.32). The “Western” pattern consisted of high intakes of sweets (0.75), sweetened beverages (0.71), added sugars (0.63), fast food (0.53), eggs (0.40), and offals (0.31) (Table 2).

**Table 2:** Factor loading matrix of the derived dietary patterns (*n* = 242)

Food groups	Diverse dietary pattern	Western dietary pattern
Fruits	0.63	0.21
Vegetables	0.61	–
Mixed dishes	0.59	–
Meat	0.54	
Dairy	0.49	0.19
Grains	0.48	0.32
Legumes and nuts	0.46	–
Fats and oils	0.32	0.25
Hot beverages	0.24	–
Sweets	–	0.75
Sugar-sweetened beverages	–	0.71
Added sugars	–	0.63
Fast food	0.23	0.53
Eggs	–	0.40
Offals	–	0.31
%Total variance explained	15.9	15.3

Factor loading < 0.2 were removed for simplicity

Pearson’s correlation coefficients for the associations between the scores of the derived patterns and nutrients are shown in (Table 3). The scores of the “Diverse” and the “Western” patterns were both positively correlated with energy intake (*r* = 0.58 and *r* = 0.60, *p* < 0.01). The “Diverse” dietary pattern scores were positively associated with proteins (*r* = 0.17, *p* < 0.01), while those of the “Western” pattern were negatively associated (*r* = − 0.36, *p* < 0.01). Higher scores of the “Diverse” pattern were associated with lower intakes of carbohydrates (*r* = − 0.15, *p* < 0.05), and higher intakes of omega 3 fatty acids (*r* = 0.30, *p* < 0.01), fibers (*r* = 0.3, *p* < 0.01), and all micronutrients considered in this study (folate, vitamin D, sodium, iron and calcium) (Table 3). The scores of the “Western” dietary pattern were directly correlated with intakes of saturated fatty acids (*r* = 0.28, *p* < 0.01), cholesterol (*r* = 0.15, *p* < 0.05), and folate (*r* = 0.19, *p* < 0.01) (Table 3).

**Table 3:** Correlation between the scores of the derived dietary pattern and energy and energy-adjusted nutrient intake (*n* = 242) ^ǂ^

	Diverse dietary pattern	Western dietary pattern
Energy	0.58^**^	0.60^**^
Proteins	0.17^**a^	−0.36^**b^
Carbohydrates	−0.15^*^	0.05
Total fat	0.10	0.11
Monounsaturated fatty acids	0.09	0.06
Saturated fatty acids	0.01	0.28^**^
Polyunsaturated fatty acids	0.08	0.01
Omega 3 fatty acids	0.30^**^	−0.04
Cholesterol	0.13^*^	0.15^*^
Trans fatty acids	−0.03	−0.07
Fibers	0.30^**^	−0.07
Folate	0.23^**^	0.19^**^
Vitamin D	0.23^**^	0.04
Sodium	0.19^**^	−0.04
Iron	0.16^*^	0.03
Calcium	0.24^**^	0.01

^*^Significant at *p* < 0.05, ^**^ Significant at *p* < 0.01^ǂ^ Energy adjusted nutrient intake was calculated using the residual method as described by Willett [31], ^a,b^ Coefficients with different alphabetical superscripts are significantly different at *p* < 0.05

Table 4 displayed the results of the multiple multinomial logistic regression analyses for the associations of the two derived dietary patterns with GWG and GWGR. The multiple regression analyses included, in addition to the pattern scores –expressed as quartiles- the following covariates: mother’s age, energy intake, parity, pre-pregnancy BMI, and GDM. Subjects belonging to the 4th quartile of the “Diverse” dietary pattern has significantly lower odds of insufficient GWG (OR: 0.24, 95% CI: 0.06–0.97) and GWGR (OR: 0.28, 95% CI: 0.09–0.90), as compared to those belonging to the 1st quartile of this pattern. On the other hand, subjects belonging to the 4th quartile of the “Western” dietary pattern had four times the odds of excessive GWG (OR: 4.04, 95% CI: 1.07–15.24) and GWGR (OR:4.38, 95% CI:1.28–15.03).

**Table 4:** Multinomial logistic regression coefficients for the association of the derived dietary patterns and GWG and GWGR (*n* = 242).^ab^

	n	GWG	GWGR	GWG		GWGR	
		mean ± SD	mean ± SD	Insufficient	Excessive	Insufficient	Excessive
Diverse pattern							
Q1	60	11.99 ± 8.28	0.48 ± 0.38	1	1	1	1
Q2	61	11.88 6.36	0.49 0.30	0.33 (0.09–1.17)	0.38 (0.12–1.22)	1.27 (0.45–3.61)	1.26 (0.45–3.51)
Q3	61	11.42 ± 6.28	0.48 ± 0.29	0.33 (0.09–1.21)	0.40 (0.12–1.31)	0.40 (0.15–1.12)	0.47 (0.17–1.27)
Q4	60	12.41 ± 8.08	0.52 ± 0.38	**0.24 (0.06–0.97)**	0.40 (0.11–1.43)	**0.28 (0.09–0.90)**	0.34 (0.11–1.07)
Western pattern							
Q1	61	10.38 ± 6.44	0.45 ± 0.30	1	1	1	1
Q2	59	12.65 ± 7.89	0.52 ± 0.33	0.95 (0.29–3.10)	2.01 (0.71–5.72)	0.97 (0.37–2.58)	1.85 (0.72–4.71)
Q3	62	10.84 ± 6.18	0.47 ± 0.34	1.43 (0.46–4.48)	1.67 (0.57–4.84)	1.68 (0.63–4.50)	1.52 (0.55–4.18)
Q4	60	13.86 ± 8.03	0.55 ± 0.34	1.73 (0.4–7.55)	**4.04 (1.07–15.24)**	2.29 (0.65–8.07)	**4.38 (1.28–15.03)**

^a^Regression models were adjusted for mother’s age, parity, energy intake, pre-pregnancy BMI, and GDM

^b^Adequate GWG and GWGR categories were considered as reference

The results of the multiple linear regression analyses examining the associations of the sociodemographic characteristics with the derived patterns showed that women older than 30 years, Arabs and with an income greater than 10,000 AED had significantly higher scores of the “Diverse” pattern (ß = 0.42, *p* = 0.01; ß =0.40, *p* = 0.02; ß =0.54, *p* = 0.001, respectively). No associations were observed between the scores of the “Western” pattern and any of the sociodemographic characteristics studied (Data not shown).

## Discussion

This study examined dietary patterns and their associations with GWG among participants of the MISC cohort. The findings revealed two main dietary patterns in the study population: “Diverse” and “Western” patterns. The “Diverse” pattern was characterized by high intakes of fruits, vegetables, mixed dishes, meat, dairy, grains and legumes, and nuts. On the other hand, the “Western” pattern consisted of sweets, sugar-sweetened beverages, fast foods, and added sugars. A common finding to most studies investigating dietary patterns is the identification of a western pattern in addition to other ‘ethnic/traditional/or Prudent’ patterns [32–37]. Though the foods constituting these patterns would vary from one study to the other, a few characteristic foods/food groups are repeatedly reported as defining each of these two patterns. More specifically, for the western dietary pattern, these foods/food groups are meat, processed meat and poultry, refined grains, sweets, desserts, fast food, snack foods, soda, and sweetened beverages; whereas for the prudent dietary patterns, these foods are fruit, vegetables, whole grains, fish and seafood, legumes, poultry, olive oil, nuts, seeds, and fat-free and low-fat dairy. It is important to note that the traditional pattern presents more heterogeneity across the literature since, as its name depicts, is context and country/region-specific [38]. The coexistence of the western with traditional dietary patterns is considered a hallmark of the nutrition transition, taking place in many countries around the globe [39]. Due to changes in lifestyle and food availability, many countries have been experiencing shifts in their dietary intakes, whereby the traditional diets are slowly eroding to be replaced by more westernized types of diets [40]. In fact, a review of studies investigating dietary patterns in the Middle East and North African (MENA) region showed that the western and traditional patterns were the most predominant among adults [39]. Within the MENA, countries of the Arabian Gulf, including the UAE, were particularly vulnerable to the nutrition transition, given the rapid shifts in these countries from a traditional semi-urbanized life to a modern and urbanized society after the major discoveries of oil in the 1960s (International Monetary Fund 2009). The nutrition transition in the UAE has been concomitant with an epidemiological transition of diseases, whereby rates of obesity, diabetes, cardiovascular disease (CVD), and many types of cancer have been on the rise in the country [41]. Therefore, it became eminent to examine the role of this nutrition transition, mainly the gradual erosion of the traditional diet and the increased adherence to the western pattern, on the etiology of these diseases and their risk factors among many population groups, including pregnant women [42, 43].

The results of this study showed that, among pregnant women in the UAE, a higher adherence to the “Western” dietary pattern was associated with higher odds of excessive GWG and GWGR, whereas a higher adherence to a “Diverse” dietary pattern led to lower odds of excessive GWG and GWGR. These findings are in line with previous research examining the effect of dietary patterns on weight gain during pregnancy. For instance, a recent study by Maugeri et al. [2] reported a positive association between adherence to a western diet (characterized by high intakes of red meat, fries, dipping sauces, salty snacks and alcoholic drinks) and excessive GWG among women participating in the ‘Mamma & Bambino’ cohort. Similar associations of the western dietary pattern with excessive GWG were reported among urban Black South African women [17], women participating in the Generation R study in the Netherlands [14], and among Finnish women [44]. Western dietary patterns are usually more energy-dense [45]. In fact, in this study, the scores of this pattern had a significantly higher correlation with energy intake compared to the “Diverse” pattern. Results stemming from both observational and interventional studies showed that women who consumed higher levels of energy had a higher weight gain as compared to women with lower energy consumption levels [14, 46]. Furthermore, in this study, the high consumption of sugars, whether in the form of added sugars, sweets, or sugar-sweetened beverages, in the “Western” dietary pattern could explain the association of the latter with GWG. A recent review of the effects of consuming sugars on maternal and child health found that high sugar consumption during pregnancy may contribute to excessive GWG, and consequently increase the risk of the development of pregnancy complications, including GDM, preeclampsia and preterm birth [47]. Fast-food consumption, a component of the western dietary pattern in most studies including this study, has also been linked to higher weight gain among pregnant women [48]. In addition to being energy-dense, fast foods are usually ultra-processed and contain a large amount of saturated and trans fatty acids [49–51]. A study conducted among United States (US) pregnant women showed that a higher percentage of energy intake from ultra-processed foods was associated with and may be a useful predictor of excessive GWG [52]. The evidence of the direct effect of the consumption of a western dietary pattern on excessive GWG calls for public health interventions to limit the adherence to this pattern and encourage the consumption of alternative patterns that are related to better health outcomes among pregnant women.

To that end, the findings of this study highlighted a protective effect of adherence to the “Diverse” dietary pattern against insufficient GWG, the latter being associated with preterm delivery and SGA infants [53]. Similar to those findings are those of Maugeri et al. from the ‘Mamma & Bambino’ cohort, who showed that adherence to the “Prudent” dietary pattern, characterized by high intakes of boiled potatoes, cooked vegetables, legumes, pizza, and soup was positively associated with GWG among underweight pregnant women and negatively among overweight and obese individuals [2]. On the other hand, Shin et al., [54] showed that a “Mixed” dietary pattern, characterized by high intakes of meat, dairy products, fruits, vegetables, potatoes, nuts, and sweets, may be associated with higher odds of insufficient GWG among pregnant women participating in the National Health and Nutrition Examination Survey (NHANES) 2003–2006. Such a discrepancy in the associations of a varied dietary pattern with insufficient GWG could be a result of the heterogeneity of what constitutes this dietary pattern among the different studies. In this study, the effect of the “Diverse” dietary pattern on the odds of insufficient GWG could be explained by the fact that adherence to a dietary pattern that consists of a variety of food groups may provide a balanced dietary intake of energy, macro, and micronutrients, and consequently lowers the odds of falling below the recommended levels of GWG, while not affecting the odds of excessive GWG [55]. In fact, foods making up the “Diverse” pattern (fruits, vegetables, mixed dishes, meat, and dairy) are rich sources of vitamins, minerals, fibers, and antioxidants. These nutrients have a positive role in the stimulation of the immune system, cholesterol synthesis, antioxidant defense, as well as modulating hormone metabolism [56].

The observed effects of dietary patterns on GWG and GWGR in this study are important especially when considered in light of recent literature revealing their significant associations with inflammation. In fact, findings from the Project Viva, a pre-birth cohort in Massachusetts, showed that consumption of a pro-inflammatory diet during pregnancy was associated with an increase in maternal systemic inflammation which could be linked to impaired fetal growth and breastfeeding failure [57]. Furthermore, a study on 671 pregnant women showed that excessive GWG, as well as high intake of western like diets were associated with higher concentrations of inflammatory factors such as high-sensitivity C-reactive protein, interleukin-8 and serum amyloid A [58]. Interventions aiming to examine the effects of lifestyle changes including the consumption of healthier diets on maternal metabolic and inflammatory markers showed significant reductions in these markers [59]. Low-grade systemic inflammation has been implicated in pregnancy complications such as preeclamsia and gestational diabetes [60]. In addition, higher levels of inflammatory markers during pregnancy are suspected to be harmful to the fetus by potentially increasing the risks of many immune diseases in childhood such as asthma and allergies [61].

While the “Western” pattern was not associated with any of the sociodemographic variables, the findings of this study showed that older women, non-Emirati Arab women, and those with higher income are more adherent to the “Diverse” dietary pattern. The results are in line with earlier studies showing that adherence to healthy dietary patterns is higher among older women, with a higher educational status and belonging to higher socioeconomic status [62, 63]. Such findings suggested that younger women and those with low socioeconomic status need specific dietary intervention programs due to the increase in nutritional demands during pregnancy.

The findings of this study also highlighted a considerable prevalence of excessive (57.4%) and insufficient GWG (24.4%), with only 18.2% gaining adequate weight. These estimates are comparable to those reported by other studies in the region. For instance, the MINA cohort conducted in two Arab countries Lebanon and Qatar, reported recently that only 30.2% of women had adequate GWG, while 25.7 and 44.1% had insufficient and excessive GWG, respectively [64]. The observed suboptimal rates of GWG are alarming especially in light of their negative health implications for both the mother and infant. With excessive GWG, mothers tend to have an increased risk of GDM, hypertensive disorders of pregnancy, cesarean section, postpartum weight retention, in addition to a lifelong risk of chronic diseases later on in life for the mother [64–66]. On the other hand, women gaining insufficient weight during their pregnancy are at an increased risk of delivering preterm birth and SGA [3, 67]. Furthermore, accumulating evidence suggested that both excessive and insufficient GWG increase the risk for obesity and its associated cardiometabolic health consequences for the infant [67–69]. The aforementioned discussion for the significant effect of GWG on health of the mother and child further highlights the importance of investigating the determinants of GWG and hence underscores the findings of the study with regards to the association of various dietary patterns with GWG among pregnant women.

The findings of this study ought to be considered in light of a few limitations. First, the pre-pregnancy BMI and GWG data were obtained from the participants’ medical records. Although standards techniques and procedures of anthropometric assessment are followed in the clinics and health centers from which the study participants were recruited, it is arguable that random errors in assessment could have occurred. Second, given that the sociodemographic and dietary intake data were obtained in an interview setting, the possibility of an interview bias or a social desirability bias could not be ruled out. However, every effort was exerted to train the field workers on the use of standard interviewing techniques and avoid leading questions. Third, the limitation inherent to the use of the principal component analysis (PCA) ought also to be considered, mainly the subjectivity in the grouping of the food items, in deciding on the number of factors to retain as well as the naming of the patterns [70].

## Conclusion

The findings of this study presented the main dietary patterns prevalent among pregnant women living in the UAE and characterized the association of these patterns with weight gain and weight gain rate during pregnancy. The two patterns were the “Diverse”, characterized by intake of fruits, vegetables, and mixed dishes and the “Western”, consisting of sweets and fast foods. These dietary patterns were differentially associated with GWG and GWGR. More specifically, adherence to the “Diverse” pattern was found to decrease the odds of insufficient GWG and GWGR, while that of the “Western” pattern was associated with higher odds of GWG and GWGR. Such findings suggested potential health risks for infants and their mothers, particularly in an oil-producing country that is facing a tide of nutrition transition resulting in one of the highest rates of obesity and related diseases worldwide [71–74]. The results of this study emphasized the need for the development of context-specific interventions to halt the nutrition transition and improve dietary quality among pregnant women living in the UAE. Such interventions would not only decrease the risk of excessive GWG during pregnancy but will also improve maternal and infant outcomes.

## Supplementary information

**Additional file 1.** Food groups and the corresponding items included in the dietary patterns analysis.

## Data Availability

The datasets used and/or analyzed during the current study are available from the corresponding author on reasonable request.

## Abbreviations

GWG: Gestational Weight Gain
LGA: Large for Gestational Age
SGA: Small for Gestational Age
UAE: United Arab Emirates
GWGR: Gestational Weight Gain Rate
MISC: Mother-Infant Study Cohort
GDM: Gestational Diabetes Mellitus
AED: Arab Emirates Dirham
BMI: Body Mass Index
PPAQ: Pregnancy Physical Activity Questionnaire
METs-min: Resting Metabolic Rate for an Activity Multiplied by the Minutes Performed
FFQ: Food Frequency Questionnaire
NICE: National Institute for Health and Care Excellence
WHO: World Health Organization
IOM: Institute of Medicine
PCFA: Principal Component Factor Analysis
KMO: Kaiser–Meyer– Olkin
MENA: Middle East and North Africa
CVD: Cardiovascular Disease
US: United States
NHANES: National Health and Nutrition Examination Survey
PCA: Principal Component Analysis
