# Supplementary material for: Dietary patterns and their associations with gestational weight gain in the United Arab Emirates: results from the MISC cohort
Source: Nutr J. 2020 Apr 21;19:36. doi: 10.1186/s12937-020-00553-9 (PMC7175557; doi:10.1186/s12937-020-00553-9)
Supplement: Supplementary file 1 — Additional file 1. Food groups and the corresponding items included in the dietary patterns analysis. [file 12937_2020_553_MOESM1_ESM.docx]

**Additional file 1.** Food groups and the corresponding items included in the dietary patterns analysis

| Food groups | Food items |
| --- | --- |
| Fruits | Canned fruits, Dried fruits, Fresh fruits, Fruit Juices, Fresh |
| Vegetables | Vegetables, canned (mixed), Vegetables, raw, Salad, green, Salad (Greek), Potato Boiled/Bake |
| Mixed dishes | Soup, Vegetables, Soup, Chicken, Eggplant, zucchini, cabbage, stuffed with rice &amp; meat, Grape leaves stuffed with rice &amp; meat , Majbouse meat,/chicken, Salouneh, yakhneh (Mloukieh, Bamieh, peas), Hreese, Markouka |
| Meat | Meat ( Lamb, beef), Poultry, fish ( Canned, cooked, fried) |
| Dairy | Milk and milk beverages (skim, low fat, whole), cheese, labneh Yogurt ( Skim, Low fat, whole) |
| Grains | Bread, Irani bread, Tishbati, Kaak, Toast crackers, breakfast cereals, rice, burghol, pasta noodles |
| Legumes and nuts | Legumes (Beans, Chickpeas, Fava Beans, Lentils, Seeds) and nuts |
| Fats and oils | Olive oil , vegetable oils , butter, Mayonnaise, Tahini, olives |
| Hot beverages | Coffee instant, Nescafe, Turkish coffee, Tea |
| Sweets | Cakes and pastries, Biscuits, Croissant, Doughnuts, Ice Cream (Regular, low fat)  ,Pudding (custard, mhalabiye) ( regular, low fat), Arabic sweets, Khabissa, Lukaimat,, khabisa, Assideh |
| Sugar sweetend beverages | Fruit Juice canned, Soda |
| Added sugars | Chocolate spread, Jam, Sugar, granules, Sugar derivatives (molasses, halawa, honey) |
| Fast food | Pies( Fatayer spinach, sambousek) Mamaeesh, Pizza, Falfel, French fries |
| Eggs | Eggs ( boiled, fried ) |
| Offal | Organ meats (liver, heart, brain, etc_ |
